# Crotoxin Modulates Events Involved in Epithelial–Mesenchymal Transition in 3D Spheroid Model

**DOI:** 10.3390/toxins13110830

**Published:** 2021-11-22

**Authors:** Ellen Emi Kato, Sandra Coccuzzo Sampaio

**Affiliations:** 1 Laboratory of Pathophysiology, Butantan Institute, São Paulo 05503-900, Brazil; ellen.kato@esib.butantan.gov.br; 2 Department of Pharmacology, Institute of Biomedical Sciences, University of São Paulo, São Paulo 05508-060, Brazil

**Keywords:** crotoxin, epithelial–mesenchymal transition, spheroid model, tumor stroma

## Abstract

Epithelial–mesenchymal transition (EMT) occurs in the early stages of embryonic development and plays a significant role in the migration and the differentiation of cells into various types of tissues of an organism. However, tumor cells, with altered form and function, use the EMT process to migrate and invade other tissues in the body. Several experimental (in vivo and in vitro) and clinical trial studies have shown the antitumor activity of crotoxin (CTX), a heterodimeric phospholipase A2 present in the *Crotalus durissus terrificus* venom. In this study, we show that CTX modulates the microenvironment of tumor cells. We have also evaluated the effect of CTX on the EMT process in the spheroid model. The invasion of type I collagen gels by heterospheroids (mix of MRC-5 and A549 cells constitutively prepared with 12.5 nM CTX), expression of EMT markers, and secretion of MMPs were analyzed. Western blotting analysis shows that CTX inhibits the expression of the mesenchymal markers, N-cadherin, α-SMA, and αv. This study provides evidence of CTX as a key modulator of the EMT process, and its antitumor action can be explored further for novel drug designing against metastatic cancer.

## 1. Introduction

Events of metastasis begin with epithelial–mesenchymal transition (EMT), whereby epithelial cells lose their inherent characteristics, including apicobasal polarity, and acquire invasive and infiltrating mesenchymal properties [1,2,3,4]. The EMT program involves the loss of expression of E-cadherin. In contrast, the expression of N-cadherin is switched on along with upregulation of other mesenchymal markers, including vimentin, fibronectin, and metalloproteases [5,6,7,8,9]. Cancer-associated fibroblasts (CAFs), activated fibroblasts commonly found in the tumor microenvironment, are characterized by higher expression of myofibroblastic markers, including α-smooth muscle actin (α-SMA). CAFs are among the predominant cells within solid tumors and secrete several soluble growth factors, including transforming growth factor-β1 (TGF-β1), interleukin-6 (IL-6), fibroblast growth factor (FGF), platelet-derived growth factor (PDGF), stromal cell-derived factor-1 (SDF-1), and hepatocyte growth factor (HGF). Some of these growth mediators can induce EMT in carcinoma cells [10,11,12].

The majority of data that highlight the importance of EMT in carcinogenesis have been obtained using in vitro cell line models. Over the last decade, three-dimensional (3D) cancer cell culture systems in vitro have been developed to understand the interactions and crosstalk between cell–cell and cell–matrix that drives tumor progression [13,14,15]. The activation of EMT is mostly studied in various cancer spheroid models [16]. Therefore, the development of novel drugs that can inhibit the onset of EMT is one of the goals of cancer research. In this direction, several compounds derived from animal venoms are considered critical scientific tools. Firstly, animal venoms have practical therapeutic applications as they provide structural templates for the development of new drugs. Secondly, studies on animal venoms have contributed significantly to understanding the regulatory mechanisms guiding cell functions under normal and pathophysiological conditions, such as cancer [17].

Snake venoms are complex mixtures of bioactive molecules [17,18,19]. Phospholipase A2 enzymes (PLA2; EC 3.1.1.4) are among the most well-characterized components of known snake venoms. Among these, crotoxin (CTX), the major toxin from *Crotalus durissus terrificus* venom, is a heterodimer comprised of a basic subunit (CB), responsible for the phospholipase activity, and neurotoxic and myotoxic properties of the molecule. The CB subunit is associated non-covalently with crotapotin, an acidic, non-enzymatic peptide (CA). The CTX complex is a potent neurotoxin, while isolated subunits present low lethality [20,21,22,23,24,25]. Sixteen CTX isoforms were identified, resulting from a random combination of four CA isoforms (CA1, CA2, CA3, and CA4) and four CB isoforms (CBa2, CBb, CBc, and CBd) [22]. These combinations between the isoforms determine the formation of different complexes, responsible for the different pharmacological and biological properties reported for CTX [21]. Several studies have shown that CTX has in vivo and in vitro anti-inflammatory, immunomodulatory, and antitumoral properties [26,27,28,29,30,31]. Cura and colleagues (2002) suggested that CTX may have greater selectivity on solid tumors since CTX could inhibit in vivo growth of Lewis lung carcinoma and MX-I human mammary carcinoma. However, it has low antitumor activity against HL-60 leukemia cells [32]. Recent studies have demonstrated that CTX not only inhibits tumor growth but also modulates stromal cells in the tumor microenvironment, such as the reprogramming of endothelial cells and macrophages, thus exhibiting an antiangiogenic phenotype [26,28,29,33].

Based on the properties mentioned above of CTX on the tumor microenvironment we also present, herein, the ability of this toxin to modulate EMT. In this study, we show for the first time, the modulatory effect of CTX on EMT markers in the 3D-spheroid model composed of tumor cells and fibroblasts. CTX is a promising target for the future development of anti-metastasis therapeutics.

## 2. Results

### 2.1. Effect of CTX on MRC-5 Cell Differentiation with Different Stimulatory Factors under 2D Condition

**Figure 1:**
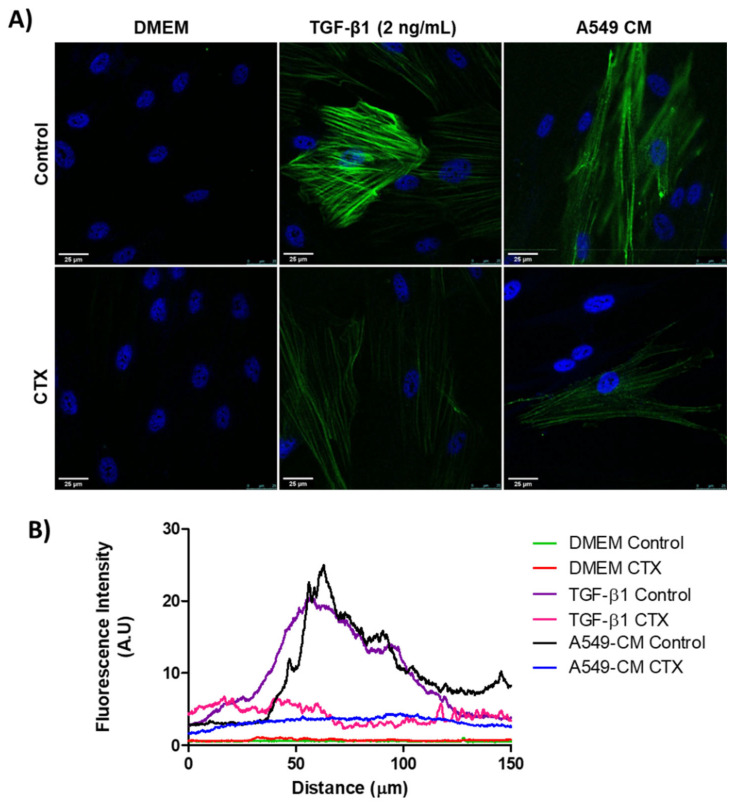
Myofibroblast differentiation with various stimulatory factors. (**A**) Representative immunofluorescent images of MRC-5 cells pretreated with CTX (12.5 nM) for 2 h and then incubated in DMEM (with 10% FBS), TGF-β1 (2 ng/mL), or tumor-conditioned media from A549 cells for 3 days. The control group of cells were untreated and grown in DMEM with 10% FBS only. (**B**) Quantification of α-SMA fluorescence intensity. Green fluorescence indicates α-SMA-containing stress fibers and blue fluorescence indicates the nuclei. Scale bar = 25 µm. The data are presented from three independent experiments.

We confirmed the expression of α-SMA in differentiating myofibroblasts through immunostaining. MRC-5 cells, previously incubated with 12.5 nM CTX, were cultured in the presence of TGF-β1 (2 ng/mL) or conditioned medium (CM) from A549 cells for 3 days. There was an undetectable level of α-SMA in both control and CTX-treated unstimulated MRC-5 cells (cultured in DMEM with 10% FBS only). We found that TGF-β1 and tumor-CM induced higher α-SMA expression in untreated than in CTX-treated MRC-5 cells (Figure 1A,B). Under the same experimental condition, Calu-3-CM induced α-SMA expression in MRC-5 cells, while in the presence of CTX this marker was undetectable (Figure S1).

### 2.2. CTX Does Not Interfere in the Spheroid Formation and Prevents Non-Spheroid from Forming Cells

**Figure 2:**
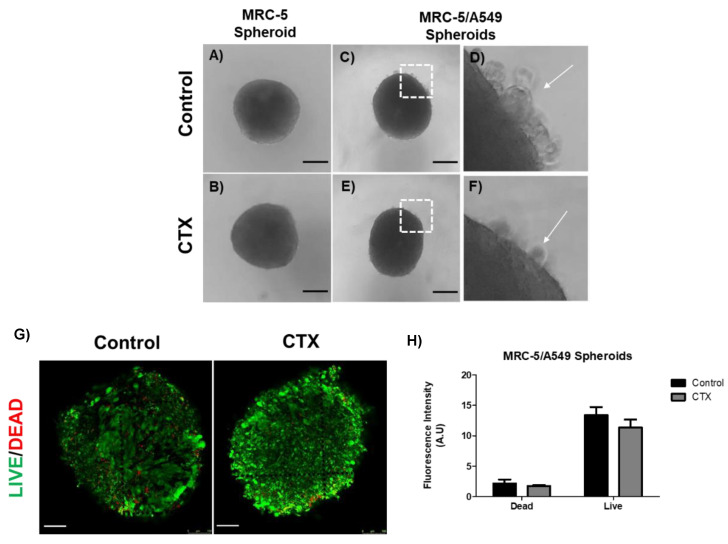
Spheroid formation by MRC-5 cells alone or in combination with A549 tumor cells. (**A**) MRC-5 single spheroid formation by the hanging drop method. (**B**) MRC-5 single spheroid constitutively formed in the presence of 12.5 nM of CTX. (**C**) MRC-5/A549 spheroid formation by the hanging drop method. (**D**) A small number of cancer cells did not incorporate into the cell aggregates (white arrow) (**E**) MRC-5/A549 spheroid constituted in the presence of 12.5 nM CTX—after 24 h, cell aggregates formed compact structures. (**F**) A small number of cancer cells did not incorporate into the cell aggregates (white arrow). Images obtained from inverted microscope 4X. (**E**) Live (green)/Dead (red) image of MRC-5/A549 spheroids. Scale bar = 100 µm. (**F**) Quantitative analysis from live/dead assay measured by relative fluorescence intensity. All the data presented here are from three independent experiments.

The human adenocarcinoma cell line A549 was mixed with MRC-5 cells in a ratio of 1:4 for spheroid formation by the hanging drop method. We observed regular round-shaped spheroids composed only of MRC-5 cells (Figure 2A); the presence of CTX did not affect the spheroid formation (Figure 2B). On the other hand, A549 cells were unable to aggregate to form a spherical structure due to weak intercellular interactions (data not shown). When A549 cells were mixed with MRC-5 cells, compact spheres were observed with a small group of tumor cells around them. These tumor cells were unable to integrate into the spheres (Figure 2C,D). Interestingly, there was a drastic reduction in tumor cells around the spheroids formed by the CTX treated MRC-5 cells (Figure 2E,F). On the other hand, MRC-5/Calu-3 spheroids were less compact compared to MRC-5/A549 spheroids (Figure S2A,B), while in the presence of CTX, fewer tumor cells were observed around the spheroid (Figure S2C,D). In addition, live/dead staining of MRC-5/A549 cells in spheroids confirmed that CTX did not affect cell viability (Figure 2E,F).

### 2.3. Spheroid Cell Invasion of Collagen Gel

**Figure 3:**
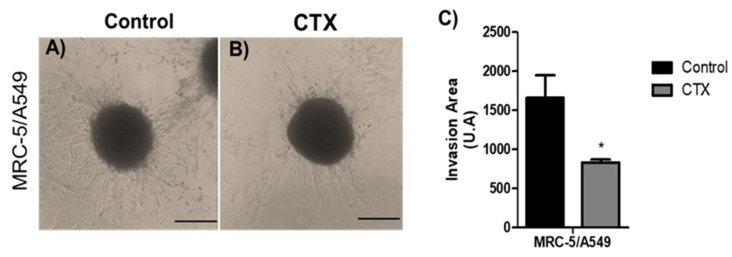
Invasion area of spheroids in 3D collagen gels. Representative images are of invasion into type I collagen gel (1.2 mg/mL) (**A**) by cells of MRC-5/A549 spheroids or (**B**) MRC-5/A549 constituted with 12.5 nM of CTX. Cell invasion was photographed under phase-contrast microscopy at 48 h. (**C**) Cell invasion area was measured and analyzed on ImageJ software. * *p* < 0.05 compared to the control group. The data presented here are from three independent experiments (*n* = 5).

To analyze the effect of CTX on the invasive phenotype, MRC-5/A549 spheroids were embedded into polymerized collagen type I gels for up to 48 h. Invading cells from MRC-5/A549 spheroids were elongated and spindle-shaped (Figure 3A). Spheroids constituted in the presence of CTX showed a 50% reduction in the invaded gel region (Figure 3B,C). On the other hand, the invasion distance of MRC-5/Calu-3 spheroids was shorter and preserved cell–cell interaction compared to MRC-5/A549 spheroids, while in the presence of CTX, the invasion area was reduced by invading cells (Figure S3).

### 2.4. Effect of CTX on the Expression of EMT Markers in Composite Spheroids

**Figure 4:**
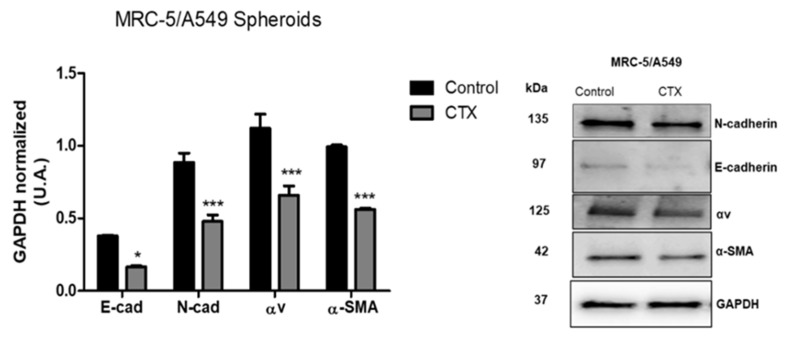
Expression of EMT-associated proteins. Quantitative analysis of expression of EMT markers. After three days in culture, MRC-5/A549 and MRC-5/Calu-3 spheroids were lysed, and Western blot analyses were conducted for E-cadherin, N-cadherin, α-SMA, integrin subunit αv. GAPDH served as the loading control. *** *p* < 0.001 compared to the control group. * *p* < 0.01 compared to the control group. The data presented are from three independent experiments (*n* = 4).

To investigate the mechanism underlying the migration ability of MRC-5/A549 spheroids, we performed Western blot analysis for epithelial marker E-cadherin and mesenchymal markers—N-cadherin, vimentin, and α-SMA. Our results showed that after 3 days, MRC-5/A549 spheroids lost E-cadherin, but the expression of all EMT markers was elevated. MRC-5/A549 constitutively formed in the presence of CTX showed a significant reduction in mesenchymal markers, α-SMA (43%), N-cadherin (46%), and integrin αv (41%) (Figure 4). However, there was no marked difference in vimentin levels. On the other hand, at the same incubation period, MRC-5/Calu-3 spheroids presented high expression of E-cadherin and low expression of mesenchymal markers, which means that MRC-5/Calu-3 spheroids need a longer period of incubation to induce EMT. However, the presence of CTX on this spheroid inhibited only α-SMA expression compared to the control group, while there was no difference in integrin αv expression (Figure S4).

### 2.5. Effect of Crotoxin on MMP-9, MMP-13, and Cytokine Secretions in 3D Collagen Gel Matrix

**Figure 5:**
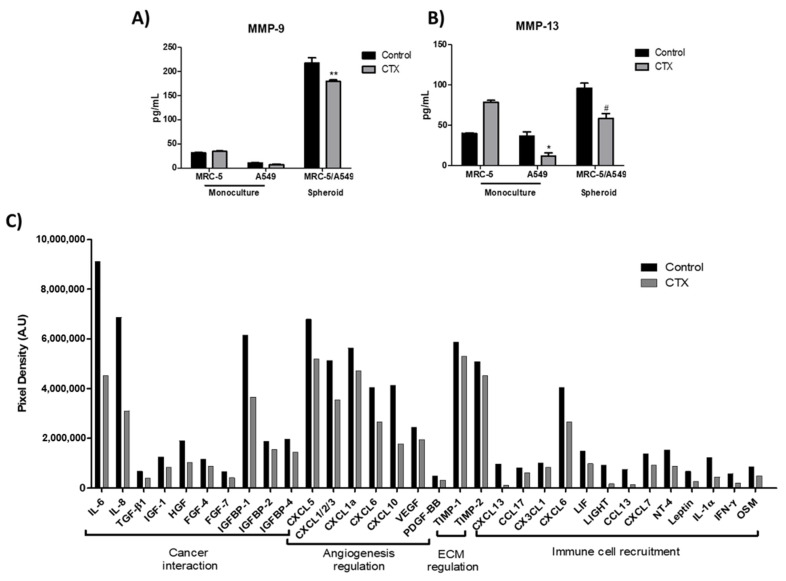
Effect of crotoxin on the production of MMP-9, MMP-13, cytokines, and chemokines in the 3D collagen gel. Monoculture of human lung fibroblasts MRC-5 and human lung adenocarcinoma cell line A549 incubated with 12.5 nM of CTX for three days; invasion of collagen gel by composite spheroids of MRC-5/A549 at 48 h. After this period, spent media were collected to quantify the volume of MMP-9 (**A**) and MMP-13 (**B**) by ELISA. * *p* < 0.05 compared to the control group. # *p* < 0.05 compared to the control group. ** *p* < 0.01 compared to the control group. (*n* = 6). In (**C**), cytokine array analysis using composite spheroid invaded gel of at 48 h. The graph indicates the decrease in the growth factors by more than 1.2-folds. A pool of *n* = 4 was used.

To determine the effect of CTX on spheroids in the 3D collagen gel, the release of matrix metalloproteinases, MMP-9 and MMP-13 (ECM-digesting enzymes), in the culture media were measured. Our results showed that a monolayer of MRC-5, grown in serum-free media, produced endogenous MMP-9; the enzyme was undetectable in media harboring A549 cells monolayer (Figure 5A). MMP-13 were produced endogenously by all cell lines. CTX did not interfere with MMP-9 release by MRC-5 cells, even though CTX repressed MMP-13 (69%) release by A549 cells (Figure 5B). Interestingly, MRC-5/A549 spheroids embedded in collagen gel promoted secretions of MMP-9 and MMP-13. However, composite spheroids treated with CTX inhibited MMP-9 and MMP-13 secretions (37% and 39%, respectively).

In addition, cytokines, chemokines, and growth factors released during the spheroid invasion of 3D collagen gel were assessed by a membrane-based cytokine array. Our results showed that the concentrations of 32 out of 80 cytokines had reduced by 1.2- to 5-fold in the culture media containing CTX-treated spheroid cells (Figure 5C). We found that CTX inhibits tumor-related cytokines, particularly IL-6, IL-8, HGF, TGF-β1, and IGFBP-1. CTX also inhibits chemokines that bind to CXCR1 and CXCR2 receptors such as CXCL5, CXCL1/2/3, CXCL1a, CXCL6, and CXCL8 (IL-8) that are involved in cancer angiogenesis and metastasis (Figure 5C).

## 3. Discussion

The present study aimed to investigate the possible modulatory effect of crotoxin, a toxin with PLA2 activity from the venom of the snake, *Crotalus durissus terrificus*, on EMT. Here, we used an in vitro spheroid model composed of human lung adenocarcinoma and human lung fibroblast cell lines. This model resembles early tumor–stroma interactions, mimicking an avascular tumor initiation step [13,34,35]. During tumor spheroid formation, fibroblasts become activated and acquire a myofibroblast-like phenotype referred to cancer-associated fibroblasts. These CAFs express contractile proteins (particularly α-smooth muscle actin), synthesize a large amount of ECM components, and secrete various matrix metalloproteinases [12,36].

In this study, we used a co-culture system to understand the reciprocal crosstalk between NSCLC tumor cell line, A549, and normal human lung fibroblast cell line, MRC-5. We found that tumor-conditioned media promoted the expression of α-SMA in MRC-5 cells, whereas CTX inhibited it. It has already been reported that the TGF-β pathway is the dominant mediator of crosstalk to initiate the process of CAF activation [37]. Previous studies have demonstrated the inhibitory effect of CTX on the functions of stromal cells such as macrophages [29] and endothelial cells [33] when co-cultivated with tumor-conditioned media. Thus, CTX impairs tumor progression. A recent study demonstrated that when human skin fibroblast cells were incubated with crude venom (CdtV) from *Crotalus durissus terrificus*, cells showed altered protrusions, formed highly polymerized actin filaments, and produced a high amount of fibronectin [38]. Based on these observations, we suggest that CTX plays varied roles in different microenvironments and may regulate the process of tissue repair.

Stadler and colleagues (2018) used different colon cancer cell lines for the spheroid formation and showed that some of the cells did not integrate into the spheroids. They hypothesized that the non-spheroid forming (NSF) cells are a subpopulation of tumor cells that had lost cell–cell adhesion properties and rendered them the ability to migrate [39]. Moreover, Sodek and colleagues (2009) correlated the ability of ovarian cancer cell lines to form compact spheroids with their migratory and invading capacity in 3D matrices. These cells exhibited myofibroblast-like features [40]. Our results showed that the presence of CTX in the composite spheroid prevented the loss of cell–cell adhesion properties of the cells and reduced the invasion area in a 3D collagen matrix.

To confirm our findings, the expression of a well-defined set of EMT-associated markers was analyzed by western blotting. Three-day-old MRC-5/A549 spheroids presented an upregulation of mesenchymal markers (such as N-cadherin, α-SMA, and integrin αv) and downregulation of E-cadherin in consensus with its rapid progression toward EMT. In contrast, as shown in the Supplementary Material, MRC-5/Calu-3 spheroids presented no alterations on EMT markers at the same experimental condition. These findings concur with a previous study that showed EMT progression in A549 and Calu-3 cells in vitro when exposed to TGF-β1 and pro-inflammatory cytokines. The authors suggest that differential cell plasticity and susceptibility to EMT may depend on tissue origin [41]. As fibroblasts become CAFs during spheroid formation, the interaction between tumor cells and CAFs leads to invasion strategies; CAFs turn into primary drivers to help tumor cells migrate by remodeling ECM and creating tracks [42]. Taken together, our data suggest that CTX significantly inhibits expression of N-cadherin, α-SMA, and integrin αv in MRC-5/A549 spheroids, which correlates with the reduced invasion area in the collagen gel. It also suggests an involvement of CTX with actin polymerization via integrin-dependent signaling pathway with subsequent impairment of migratory ability, a finding that was also observed in endothelial cells in the tumor microenvironment [33]. These findings are in agreement with a similar study conducted with PLA2 (BthTX-II) extracted from the venom of *Bothrops jararacussu*. BthTX-II displayed a weak catalytic activity and presented an inhibitory effect on the adhesion, proliferation, invasion, and migration of human breast cancer cells. It also inhibited EMT by modulating epithelial and mesenchymal markers [43]. Many studies have shown the inhibitory effect of other PLA2s on tumor cells, however the findings did not correlate the effect with the enzymatic activity of the molecule. CTX acts on focal adhesion kinases (FAK), a crucial component of integrin-mediated cell signaling in endothelial cells [33], and inhibits tyrosine phosphorylation, consequently inhibiting the activity of proteins involved in the intracellular signaling pathway of macrophages [44].

Our data suggest that CTX affected MMP-9 secretions, which significantly increased during tumor–CAF crosstalk in the spheroid model. Unsurprisingly, monocultures of A549 and MRC-5 cells released endogenous levels of MMP-9. It has been shown that high expression of MMP-9 is associated with the aggressiveness of malignant cells in solid tumors [45]. MMP-9 has also been reported to activate the bioactive form of TGF-β [46] and downregulate the expression of E-cadherin [47], thus initiating the process of EMT. Moreover, Eberlein and colleagues (2015) reported that the activation of normal fibroblast during tumor cell–fibroblast crosstalk occurs through the αvβ6/TGF-β signaling pathway [37]. We hypothesize that CTX regulates TGF-β activation since our findings showed reduced secretions of MMP-9 and αv integrin in the spheroid model. Moreover, human MMP-13 is expressed in skin fibroblasts and has a role in acute wound healing by remodeling fibrillar collagens [48]. In this study, we found an increased secretion of MMP-13 in MRC-5 monoculture incubated with CTX, suggesting its involvement in wound healing. The modulatory activity of CTX during the healing process in an inflammatory environment has already been shown in an earlier study [49]. Conversely, MMP-13 secretions were reduced in A549 monoculture in the presence of CTX as well as in the heterospheroid model. A study using NSCLC from patients showed that both MMP-9 and MMP-13 were associated with metastasis, invasion, and prognosis; MMP-13 mainly activates MMP-9 to participate in the invasion and metastasis of NSCLC [50]. This corroborates our finding of the microenvironment-dependent modulatory activity of CTX on the release of MMP-9 and MMP-13.

Cytokines released during MRC-5/A549 spheroids’ invasion of collagen gel have shown that CTX drastically inhibits chemokines that bind to the receptor CXCR1 and CXCR2, such as CXCL5, CXCL-8 (IL-8), CXCL1/2/3, CXCL1a, and CXCL6. It is well established that the CXCL5/CXCR2 and IL-8/CXCR1/CXCR2 axes contribute to carcinogenesis by promoting tumor cell proliferation, migration, and invasion, and the EMT process of many tumor cells, including NSCLC [50,51,52,53], hepatocarcinoma cells [54], and papillary thyroid carcinoma cells [55]. Additionally, CTX inhibits primary growth factors involved with EMT such as HGF [56], TGF-β1 [37,57], and VEGF [53].

## 4. Conclusions

This is the first scientific report about the modulatory effect of CTX on paracrine signaling during tumor–stroma crosstalk as it inhibits CAFs’ differentiation. In the 3D model, CTX repressed the expression of mesenchymal markers, chemokines, and growth factors associated with the EMT process. CTX also suppressed the invasion of collagen gel due to a decrease in MMP-9 and MMP-13 secretions and activities. We have provided evidence that CTX is a potential modulator of the signaling cascade involved in the progression of EMT, a critical phase that results in metastasis.

## 5. Materials and Methods

### 5.1. Crotoxin

CTX was obtained from the venom collected from several *Crotalus durissus terrificus* and provided by the Laboratory of Herpetology, Butantan Institute. The purification of this toxin was performed as described previously [58]. The purity of CTX was verified by non-reducing sodium dodecyl sulfate-polyacrylamide gel electrophoresis (12.5%) [59] and PLA2 activity was assessed by colorimetric assay using a synthetic chromogenic substrate as described elsewhere [60].

### 5.2. Cell Culture

The human non-small cell lung adenocarcinoma A549 cell line was kindly provided by Dr Durvanei Augusto Maria, Laboratory of Biochemistry and Biophysics, Butantan Institute. MRC-5 human lung fibroblast cell lines were purchased from Rio de Janeiro Bank Cells (Banco de Células do Rio de Janeiro, BCRJ code-0180) (Rio de Janeiro, Brazil). Monolayer cell was cultured in 75 cm² flasks, containing DMEM (Dulbecco’s Modified Eagles Medium) supplemented with 10% fetal bovine serum (FBS), 100 units/mL of penicillin, and 0.1 mg/mL of streptomycin (CultiLab) with 5% CO_2_ at 37 °C for 48 h. Sub-confluent cells were trypsinized with trypsin/EDTA (Gibco^®^) for subsequent use. A549 cells from passage 32–37, MRC-5 cells from passage 29–32 were used in our experiments.

### 5.3. Spheroid Preparation and Culture

Composite spheroids containing a mixture of tumor cells A549 and MRC-5 fibroblasts were prepared based on the hanging drop method as described earlier [13,35]. Sub-confluent cells were trypsinized and resuspended in DMEM with 10% FBS to a concentration of 1 × 10^6^/mL. Cell suspension of fibroblasts and tumor cells were prepared with or without 12.5 nM of CTX and then mixed at the ratio of 4:1 (4 fibroblasts per 1 tumor cell). Approximately 40 drops (25 mL/drop, 2.5 × 10^4^ cells) were dispensed onto a lid of a cell culture dish. The lid was then inverted and placed over a cell culture dish containing a culture medium for humidity. For spheroid optimization, it was added to suspension cells 0.24% of methylcellulose so that it can contribute to more compact and circular spheroid morphology. The lid was incubated for 24 h, at 37 °C, and 5% CO_2_.

### 5.4. Invasion in Three-Dimensional Spheroid Collagen Gel

Type I collagen gel (1.2 mg/mL) was prepared as described by [13]. Type I collagen gel solution (5 mL of 2X DMEM, 1 mL 10X HEPES (0.2 M, pH 8.0), and 4 mL type I collagen (3 mg/mL, PureCol, Advanced Biomatrix^®^)) were prepared and kept on ice before and during experiments. For this assay, 100 µL/well of collagen gel was added in a 48-well plate; it was allowed to polymerize for 30 min at 37 °C to form the first layer of the gel. After gel polymerization, the spheroid was embedded into the collagen gel by pipetting it into the second collagen gel layer (100 µL/well). The collagen–spheroid mixtures were then left to polymerize in the cell culture incubator, the 400 µL of serum-free DMEM media were added on top of the wells. After 48 h, the spent media were collected for ELISA assay. Cell migration from spheroids embedded in collagen gels was monitored under an inverted light microscope (Leica DMIL^®^, Wetzlar, Germany) and photographed at different time points.

### 5.5. Confocal Immunofluorescence Analysis

A monolayer of MRC-5 (2 × 10^5^/well) was grown in DMEM supplemented with 10% SFB for 24 h. Fibroblasts were then treated with CTX (12.5 nM) for 2 h and, washed with PBS and cultured in either DMEM with 1% SFB in the presence of 2 ng/mL of TGF-β1 (Peprotech, cat number 100–21C, Cranbury, NJ, USA) or conditioned media from A549 cells for three days. After this, cells were fixed in 4% paraformaldehyde for 20 min, rehydrated with PBS (3 × 10 min), permeabilized in 0.2% Triton X-100 in PBS for 10 min, and kept overnight in a blocking solution containing PBS with 1% BSA and 0.1% Triton X-100, at 4 °C. The slides were incubated for 1 h with anti-α-SMA (1:250) in PBS with 1% BSA, and 0.1% Triton X-100 at 37 °C. After three washes with PBS containing 0.05% Tween 20, the slides were incubated with Goat anti-rabbit conjugated with DyLight 549 (1:800) for 1 h at room temperature. The slides were washed twice and then incubated with DAPI (25 mg/mL; Sigma Aldrich, Saint Louis, MO, USA) for 2 min. Mounting medium was applied, and the cells were photographed using a Leica DMi8 confocal microscope (Leica Microsystems, Mannheim, Germany), equipped with a DFC 310 FX digital camera, 63× magnification with oil immersion. Images were captured with the LAS AF and processed with the ImageJ software (National Institutes of Health, Bethesda, MD, USA).

### 5.6. Western Blotting

Spheroids of MRC-5/A459 were lysed, and total cellular protein was extracted using RIPA lysis (Sigma Aldrich, R0278) with a protease and phosphatase cocktail (Sigma Aldrich, P0044; P5726; P8340). Cell lysates were then centrifuged at 12,000 rpm for 30 min at 4 °C, the supernatant containing the soluble proteins was collected and measured by the BCA protein assay (Novagen^®^, 71285). The samples containing 30 µg protein were subjected to SDS/PAGE under reducing conditions on a 4–20% gradient polyacrylamide gel (Bio-Rad, cat no. 456–1094, Hercules, CA, USA). Following electrophoresis, proteins were transferred to a nitrocellulose membrane, which was then blocked with TBS-T (20 mM Tris-HCl, 150 mM NaCl, and 0.1% Tween 20) containing 5% nonfat dry milk for 2 h. The membranes were incubated with primary antibodies: anti-E-cadherin (1:1000), anti-N-cadherin (1:1000), anti-vimentin (1:5000), anti-α-SMA (1:2000), anti-integrin αv (1:2000), and anti-GAPDH (1:1000) overnight at 4 °C (Abcam). The membrane was subsequently incubated with peroxidase-conjugated secondary antibody (anti-mouse IgG and anti-goat IgG) for 2 h. Detection was performed with an enhanced chemiluminescence kit (Thermo Scientific, Rockford, IL, USA). The signals were detected with an image acquisition system (Alliance 9.7, Uvitec, Cambridge, UK). Band intensities were measured with Image J software (NIH).

### 5.7. ELISA Assay

As mentioned earlier, spheroids’ spent media were collected for collagen invasion assay. Human MMP-9 (ab100610) and MMP-13 (ab100605) were quantified by using specific ELISA kits following the manufacturer’s instructions. ELISA Kits were purchased from Abcam Company (Cambridge, UK).

### 5.8. Human Cytokine Array Assay

The expression levels of chemokines and cytokines were analyzed using a Human Cytokine Antibody Array (C5) (RayBiotech, Inc., Norcross, GA, USA). As per the manufacturer’s instructions, antibody-embedded membranes were incubated with 1 mL of conditioned media (CM) at 4 °C overnight, followed by incubation with a biotin-conjugated detection antibody cocktail and diluted HRP-streptavidin at room temperature. Proteins were then visualized using a chemiluminescent substrate reagent. The intensity of each spot represents cytokines, which were then quantified densitometrically using ImageJ software.

### 5.9. Statistical Analysis

The GraphPad Prism software was used (version 5.0). Student’s *t*-test and one-way ANOVA (followed by Tukey’s post test) were used for comparisons, and differences were considered significant at *p* < 0.05.

## Data Availability

Not applicable.

## Supplementary Materials

The following are available online at https://www.mdpi.com/article/10.3390/toxins13110830/s1, Figure S1: Myofibroblast differentiation in Calu-3 conditioned medium. Figure S2: MRC-5/Calu-3 spheroid. Figure S3: Invasion area of MRC-5/Calu-3 spheroids in 3D collagen gels. Figure S4: Expression of EMT-related proteins.
