# Supplementary material for: Crotoxin Modulates Events Involved in Epithelial–Mesenchymal Transition in 3D Spheroid Model
Source: Toxins (Basel). 2021 Nov 22;13(11):830. doi: 10.3390/toxins13110830 (PMC8618719; doi:10.3390/toxins13110830)
Supplement: Supplementary file 1 [file toxins-13-00830-s001.zip › toxins-1443790-sup.pdf]

Supplementary Materials: Crotoxin modulates events involved in epithelial-mesenchymal transition in 3D spheroid model

Ellen Emi Kato, Sandra Coccuzzo Sampaio

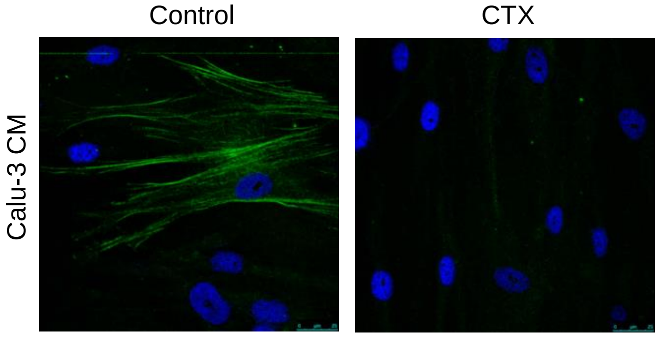

Figure S1. Myofibroblast differentiation in Calu-3 conditioned medium. Representative immunofluorescent images of MRC-5 cells pretreated with CTX (12.5 nM) for 2h and then, incubated in presence of tumor conditioned media from Calu-3 cells for 3 days. Green fluorescence indicates α-SMA-containing stress fibers expression and blue fluorescence indicates the nuclei. Scale bar = 25 μm.

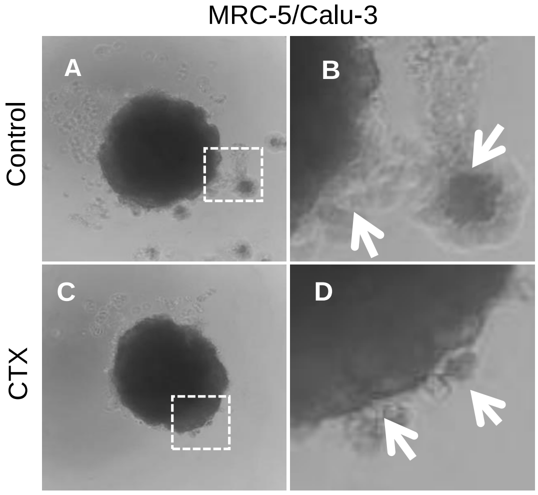

Figure S2. MRC-5/Calu-3 spheroid formation. MRC-5/Calu-3 spheroid formation was performed by hanging drop method. After 24 h cells aggregates formed compacted structures in the absence (A) or in the presence of CTX (C). A small amount of cancer cells did not incorporated into compacted cell aggregates (C and D)(arrow).

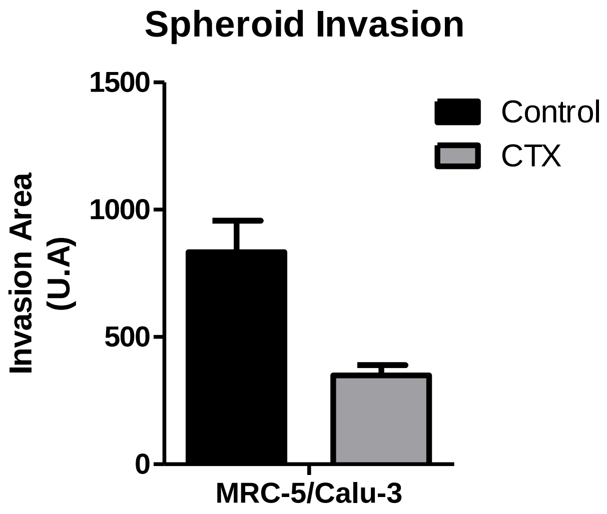
Spheroid Invasion
Invasion Area (U.A)
1500
1000
500
0
Control
CTX
MRC-5/Calu-3

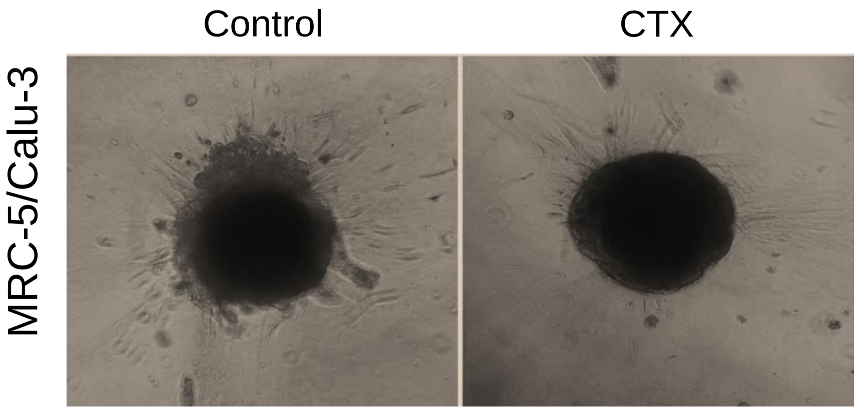

Figure S3. Invasion area of MRC-5/Calu-3 spheroids in 3D collagen gels. Spheroids MRC-5/Calu-3 constituted with 12.5nM CTX were embedded in collagen gel (1.2mg/mL) and cell invasion was photographed under phase-contrast microscopy up to 48h. Cell invasion area was measured and analyzed on ImageJ software.

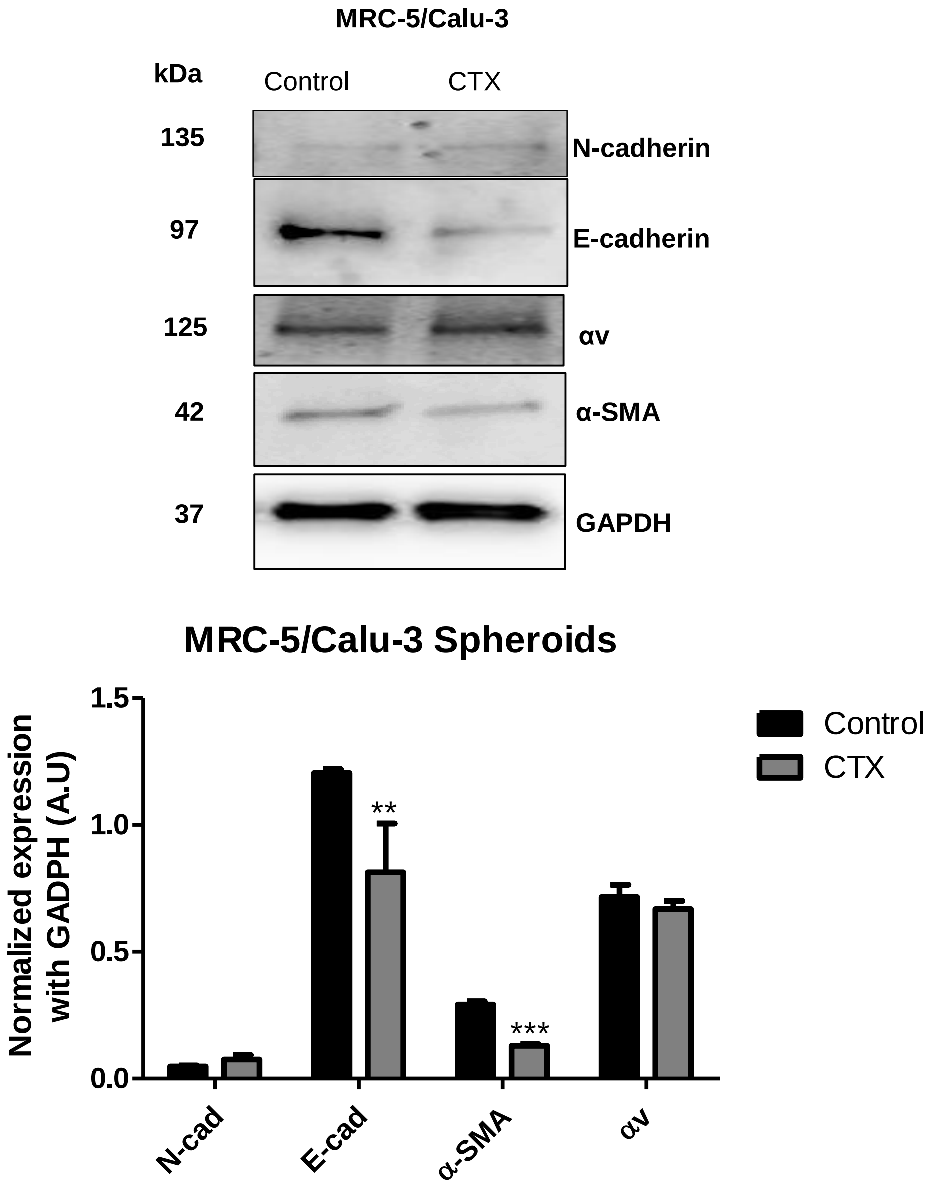

Figure S4. Expression of EMT-related proteins. After 3 days in culture, MRC-5/Calu-3 spheroids were lysated and analyzed on Western blot for E-cadherin, N-cadherin, α-SMA and GAPDH as loading control. *** $p<0.001$ compared to control group. ** $p<0.01$ compared to control group (n=4).
